# Examination of ELISA against PCR for assessing treatment efficacy against Cryptosporidium in a clinical trial context

**DOI:** 10.1371/journal.pone.0289929

**Published:** 2023-09-08

**Authors:** James T. Nyirenda, Marc Y. R. Henrion, Vita Nyasulu, Mike Msakwiza, Wilfred Nedi, Herbert Thole, Jacob Phulusa, Neema Toto, Khuzwayo C. Jere, Alex Winter, Leigh A. Sawyer, Thomas Conrad, Donnie Hebert, Crystal Chen, Wesley C. Van Voorhis, Eric R. Houpt, Pui-Ying Iroh Tam, Darwin J. Operario

**Affiliations:** 1 Malawi-Liverpool-Wellcome Trust Clinical Research Programme, Blantyre, Malawi; 2 Liverpool School of Tropical Medicine, Liverpool, United Kingdom; 3 Centre for Global Vaccine Research, Institute of Infection, Veterinary and Ecological Sciences, University of Liverpool, Liverpool, United Kingdom; 4 Emmes Corporation, Rockville, Maryland, United States of America; 5 University of Virginia, Charlottesville, Virginia, United States of America; 6 University of Washington, Seattle, Washington, United States of America; George Washington University School of Medicine and Health Sciences, UNITED STATES

## Abstract

**Background:**

*Cryptosporidium* is a gastrointestinal pathogen that presents a serious opportunistic infection in immunocompromised individuals including those living with human immunodeficiency syndrome. The CRYPTOFAZ trial, previously published, was conducted in Malawi to evaluate the efficacy of clofazimine in response to an unmet need for drugs to treat cryptosporidiosis in HIV populations. A combination of rapid diagnostic tests, ELISA, qPCR, and conventional sequencing were employed to detect *Cryptosporidium* in 586 individuals during pre-screening and monitor oocyst shedding and identify enteric co-pathogens in 22 enrolled/randomized participants during the in-patient period and follow-up visits.

**Methodology:**

Oocyst shedding as measured by qPCR was used to determine primary trial outcomes, however pathogen was detected even at trial days 41–55 in individuals randomized to either clofazimine or placebo arms of the study. Therefore, in this work we re-examine the trial outcomes and conclusions in light of data from the other diagnostics, particularly ELISA. ELISA data was normalized between experiments prior to comparison to qPCR. The amount of all identified enteric pathogens was examined to determine if co-pathogens other than *Cryptosporidium* were major causative agents to a participant’s diarrhea.

**Conclusion:**

ELISA had higher sample-to-sample variability and proved to be equally or less sensitive than qPCR in detecting *Cryptosporidium* positive samples. Compared to qPCR, ELISA had equal or greater specificity in detecting *Cryptosporidium* negative samples. Sequencing identified several *Cryptosporidium* species including *viatorum* which has never been identified in Malawi and Southern Africa. In addition to *Cryptosporidium*, enterotoxigenic *E*. *coli* was also identified as a pathogen in diarrheagenic amounts in 4 out of 22 participants.

## 1.0 Introduction

*Cryptosporidium*, the causative agent of cryptosporidiosis, is a gastrointestinal pathogen of both humans and animals spread through fecal-oral route and has a global distribution [1]. Human infections of *Cryptosporidium*, thought to be caused primarily by *C*. *parvum or C*. *hominis* [2], represent a serious opportunistic infection in immunocompromised individuals including those living with human immunodeficiency virus (HIV) [3]. Globally, prevalence of cryptosporidiosis is estimated at 7.6% [4] and amongst HIV-positive individuals, the global pooled prevalence of *Cryptosporidium* is 14%. In Sub-Saharan Africa, the estimate is 21.1% among HIV infected individuals [5].

Currently, nitazoxanide is the only US Food and Drug Administration approved drug for treatment of cryptosporidiosis and is only recommended among people with healthy immune systems [6, 7]. Given that nitazoxanide has been shown to be ineffective in HIV infected individuals and has about 56% efficacy in malnourished populations [8, 9], there is a huge unmet need for drugs to treat this disease. Clofazimine (CFZ, sold as Lamprene^®^; Novartis, Switzerland) has recently been described as effective against *Cryptosporidium in vitro*, and was able to eliminate *C*. *parvum* in a mouse model [10].

We conducted a Phase 2A clinical trial to evaluate CFZ efficacy in people with HIV presenting with persistent diarrhea (diarrhea lasting at least 14 days) due to *Cryptosporidium* in Malawi (“CRYPTOFAZ”, clinicaltrials.gov study NCT03341767 [11]). We have published the primary clinical outcomes [12] and pharmacokinetics and pharmacodymics of CFZ in treating cryptosporidiosis [13] Unfortunately, the trial was unable to demonstrate CFZ efficacy for cryptosporidiosis treatment.

Current diagnostic methods for *Cryptosporidium* include histology, microscopy, rapid immunochromatographic diagnostic tests (RDTs), enzyme linked immunosorbent assay (ELISA), and polymerase chain reaction (PCR) [14]. In developing countries, morphological identification of *Cryptosporidium* oocysts by microscopy is the most widely used method for the diagnosis due to its relatively low cost [15] and can be aided by fluorescent antibodies to increase sensitivity and specificity against non-*Cryptosporidium* antigens [16]. As a primary study objective, the CRYPTOFAZ study evaluated the reduction of *Cryptosporidium* oocyst shedding following CFZ or placebo administration using qPCR as the primary diagnostic method. However, multiple diagnostics were employed during the trial, including RDTs and ELISAs. Nucleic acid-based methods for *Cryptosporidium* detection such as PCR have increased sensitivity compared to both modified Ziehl Neelsen microscopy and antigen-based assays [17, 18]. The specificity of PCR-based methods over antibody-based diagnostics enables not only *Cryptosporidium* detection, but also subtype family identification [15, 16].

In light of a recent report indicating that certain enteropathogens, including *Cryptosporidium*, may have prolonged shedding and persistence as detected through qPCR [19]. PCR has potential to detect DNA from oocysts that are not intact and hence this may affect monitoring of oocysts shedding in a clinical trial context. However, ELISA detects intact oocysts and may not be affected by the issue of persistence or prolonged shedding. Because the (qPCR) assay is quantitative by design, qPCR was the preferred monitoring method. We were nonetheless interested in comparing to the ELISA result. Our purpose in this paper was to examine if and how data from other diagnostics used in the trial, particularly ELISA, as well as from the RDT, array card PCR and genotyping support or confound the study conclusions achieved through plate-based qPCR (PCR done using 96 well plate).

## 2.0 Materials and methods

All procedures were carried out at the Malawi-Liverpool Wellcome Trust Clinical Research Programme in Blantyre, Malawi unless otherwise indicated.

### 2.1 *Cryptosporidium* rapid diagnostic test

A point of care immunochromatographic testing kit (rapid diagnostic test, RDT), *Cryptosporidium* EZ VUE (Techlab, Blacksburg, VA, USA) was used as a screening tool according to the manufacturer’s instructions. Briefly, all reagents and freshly collected stool samples were brought to room temperature before testing. Prior to testing samples were mixed by stirring or vortexing based on consistency. Either 50μL liquid or 0.05g solid fecal sample was diluted before being exposed to a test strip. Results were read after 10 minutes and a positive result was interpreted when the control and test lines appeared and a negative result when only the control line appeared. *Cryptosporidium* RDT was performed on samples from pre-screening visit.

### 2.2 Enzyme linked immunosorbent assay

A commercial ELISA kit, *Cryptosporidium* II (Techlab) was used to detect *Cryptosporidium* antigens from samples corresponding to trial days -1 (baseline), 12, 4, 6, 19–24 days (follow up 1) and 41–55 (follow up 2) following manufacturer’s instructions. The test was carried out in batches on fecal samples previously frozen at -80°C. Samples and reagents were brought to room temperature prior to testing. Results were read at a dual wavelength of 450–620 nm using a Biochrom EZ Read 400 ELISA reader (Biochrom, Cambridge, UK) with samples having optical density (OD) of ≥0.090 considered positive and OD<0.090 was considered negative. Positive and negative controls were run within each batch of testing. ELISA data from different plates then normalized.

### 2.3 Total nucleic acid DNA extraction, plate-based qPCR, and TaqMan array card

Total nucleic acid was extracted from fecal samples using the QIAamp Fast DNA Mini Kit (QIAGEN, Hilden, Germany) with a procedure modified from that of the manufacturer as previously described [20]. Briefly, 200mg solid stool or 200μL liquid fecal samples were first mixed with InhibitEX buffer and glass beads before bead beating (Tissue Lyser II, Qiagen). Resulting lysates were heated at 95°C for 5 minutes prior to proceeding according to the manufacturer’s protocol. All samples were spiked with Phocine herpes virus (PhHV) and MS2 phage to be used as extraction controls. One extraction blank (200μL nuclease-free water as the sample) was included in each batch of extractions to monitor for contamination.

Plate-based qPCR was performed as previously described [21]. These qPCRs were carried out using the ViiA7 or QuantStudio 7 Flex Real-Time PCR instruments (Thermo Fisher, Waltham, MA, USA). Primers and probes were sourced from Integrated DNA Technologies (IDT, Coralville, Iowa, USA) and Sigma (Sigma-Aldrich, Haverhill, UK). Each qPCR run included a dilution series of known amounts of *Cryptosporidium* genomic DNA (derived from extraction of *Cryptosporidium* oocysts) and PhHV acting as positive controls and standard curves, as well as a negative control (5μL nuclease free water in place of nucleic acid extract). Plate-based qPCR was performed on samples from the pre-screening visit (regardless of *Cryptosporidium* RDT results), trial days 0–6, and the two follow-up visits.

Detection of stool pathogens at patient baseline was performed using custom-designed TaqMan Array Card (TAC) as previously described [20]. Briefly, nucleic acid extract was mixed with the AgPath-ID One-Step RT-PCR kit (Thermo Fisher) prior to application to the array card. Reaction conditions were as previously described. All TAC-PCRs were conducted on the QuantStudio 7 Flex PCR instrument.

All resulting qPCR data from both plate- and TAC-based PCR were analyzed using QuantStudio 6 and 7 Flex Real-Time PCR System Software, ver. 1.3 (Thermo Fisher), An analytical cutoff of 35 cycles was applied to the data (*i*.*e*. C_t_ values ≥35.0 were considered negative). In plate-based qPCR, *Cryptosporidium* C_t_ values were converted to a genome count through comparison to the standard curve, and then to equivalent oocyst count by dividing by 4 (4 nuclei per oocyst). This calculation was based on the assumption that all cryptosporidium oocysts in the sample came for intact oocysts whose DNA was recovered during extraction from stool samples.

### 2.4 Sequencing for subgroup determination

Further characterization of *Cryptosporidium* from baseline samples targeted the 18S rRNA and gp60 genes [22, 23] and was achieved using endpoint PCR performed at Houpt Laboratory at the University of Virginia in Charlottesville, Virginia, USA followed by commercial Sanger sequencing (Genewiz, South Plainfield, New Jersey, USA).

### 2.5 Statistical analysis

Full, reproducible R code for all analyses detailed below can be accessed from GitHub (https://github.com/mlw-stats/CRYPTOFAZ-diagnostics). While noting that the ELISA assay used in this work is designed and licensed for qualitative (positive / negative) analyses only, we explored the use of the OD measurements from this ELISA assay to quantify the amount of Cryptosporidium oocysts. For these exploratory, quantitative analyses, ELISA OD values were normalized using the positive and negative controls from each plate. Specifically:

$$OD_{normalized}=(OD_{raw}-OD_{neg\;ctrl})/(OD_{pos\;ctrl}‐OD_{neg\;ctrl})$$

For qualitative ELISA results, we determined positivity / negativity according to the manufacturer’s guidelines.

To compare qPCR C_t_ values between ELISA negative and positive samples, we used a mixed censored regression model with qPCR C_t_ as response variable, ELISA positivity as a fixed factor and participant ID as a random factor. This model accounts both for the correlated nature of the data (multiple observations for the same individual) and the fact that for negative detections with C_t_ values of 40, the only information that is known is that the Ct value is at least 40, but might have been higher had more PCR cycles been run. This model was implemented using the R package censReg [24].

Within-subject repeated measures Pearson correlation coefficient was calculated using the R package rmcorr v.0.3.1 [25, 26], while between-subject correlation coefficient was calculated using the cor.test function from the stats [27] package in R.

Statistical analyses were conducted using Microsoft Excel 2013 and R v4.0.2 [27].

### 2.6 Ethical approval

The main study was approved by the National Health Science Research Committee of Malawi (Reference 17/05/1821) and the Liverpool School of Tropical Medicine Research Ethics Committee (Reference 17–031). Approval for importation and use of the study investigational products was obtained from the Pharmacy Medicine Poisons Board of Malawi (Reference PMPB/CTRC/2A/CFZ-001). Written informed consent was obtained from the study participants before being enrolled into the trial.

## 3.0 Results

A total of 586 potential participants were screened for study enrollment, with 558 patient samples screened using both qPCR and RDT. PCR was performed on all samples regardless of RDT results, hence PCR was the primary screening tool. Nine individuals were tested only using RDT, 2 only with qPCR, and 17 potential participants were untested (Table 1). Five participants were screening failures hence they were excluded from the analysis, 21 participants (3.8%) tested positive for *Cryptosporidium* under both diagnostics and 54 (9.8%) tested positive with qPCR only. From these 75 qPCR positives, only 22 met the trial inclusion and exclusion criteria and were enrolled and randomized into the study.

**Table 1:** Participant screening results.

		qPCR (cut-off C_t_ = 35)		
		Positive	Negative	qPCR untested
RDT	Positive	21	0	0
	Negative	54	483	9
	RDT untested	0	2	17

Stool samples from potential study participants were screened for *Cryptosporidium* using both rapid immunochromatographic diagnostic tests (RDT) and plate based quantitative PCR. For screening purposes, a sample was considered qPCR-positive if the resulting C_t_ value was below 35.0. A total of 553 participants were screened using both these methods. Nine individuals were tested only using RDT, 2 only with qPCR, and 17 potential participants were untested hence these were not included in the analysis (N/A).

**Fig 1:**
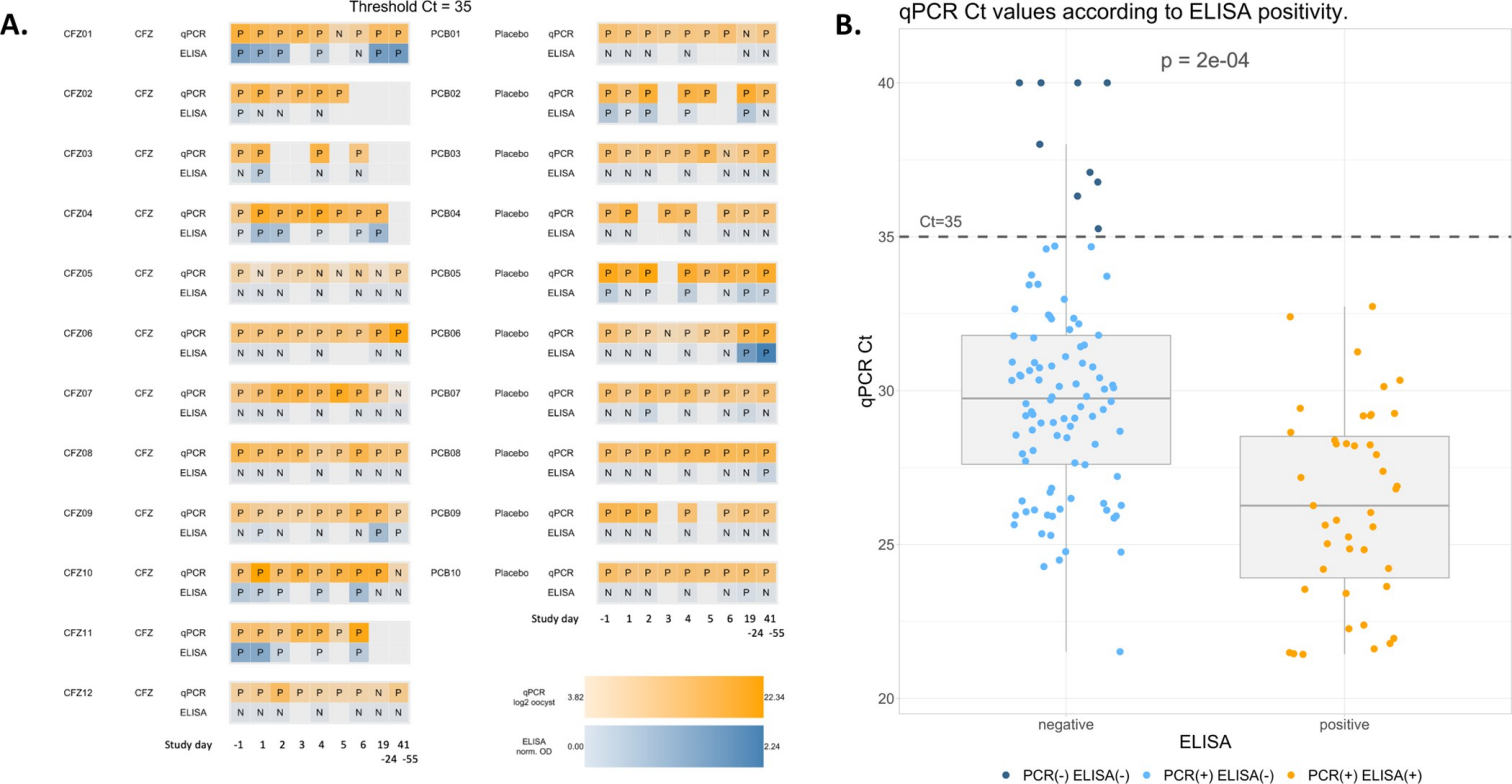
Comparison of ELISA and qPCR results. Participant stools were monitored for oocyst shedding during the inpatient period and follow-up visits using both ELISA and plate-based qPCR with a C_t_ cut-off of 35. A) This heatmap depicts the day-to-day comparison of the qPCR result (orange hues) versus the normalized ELISA OD value (blue hues) for the same sample. The more intense the coloration, the lower the qPCR C_t_ value or the higher the ELISA OD value. Quantitative PCR was conducted on all trial days, while ELISA was conducted on trial days -1, 1, 2, 4, 6, 19–24, and 41–55. B) Semi-quantitative correlation analysis of ELISA vs. qPCR. CFZ, participant randomized to the clofazimine arm of the study; PCB, participant randomized to the placebo arm of the study. P, positive; N, negative; grey square, no value/not tested.

The primary objective of CRYPTOFAZ was to evaluate whether there was a reduction in the fecal shedding of *Cryptosporidium* oocysts following the oral administration of Clofazimine or placebo control. Monitoring of the 22 enrolled participants (12 randomized to CFZ, 10 to placebo) was achieved through use of qPCR and ELISA during the in-patient period as well as two follow-up visits. Because the assay is quantitative by design, qPCR was the preferred monitoring method. We were nonetheless interested in comparing to the ELISA result. To achieve this, for quantitative comparisons, we normalized the ELISA results from different batched runs against one another (see Materials and Methods) and aligned the day-to-day results of the two diagnostics, after verifying the consistency of the qPCR runs against each other (See S1 Fig in S1 File). Fig 1A shows that on days when both methods were employed, qPCR and ELISA were moderately correlated with one another. Indeed, by within-subject repeated measured Pearson correlation coefficient, ρ = 0.38 (95% CI = [0.20, 0.53]), qPCR derived log_2_ oocyst count per gram stool and normalized ELISA ODs were moderately correlated. The between-subject Pearson correlation coefficient was similar (but with a wider confidence interval given the lower number of data points used in that calculation), ρ = 0.37 (95% CI = [-0.06, 0.69]). Of a total 141 samples tested by both methods, 52 (36.9%) had fully concordant qualitative results between the two diagnostics (Table 2). We stratified the data by qualitative ELISA result and then examined the qPCR data using a positivity cut-off of C_t_ 35. As shown in Fig 1B, samples testing positive in ELISA had a median C_t_ value of 26.3 (IQR, 23.9–28.5) which was lower compared to the median C_t_ value of 29.8 (IQR, 27.6–31.8) for those samples testing as ELISA negative. The 3.5-unit difference in values is statistically significant (p = 2e-04) and is roughly equivalent to a one-log difference in detected DNA.

**Table 2:** Cross-tabulation of qPCR and ELISA results.

		ELISA (OD Normalized)	
		Positive	Negative
qPCR	Positive	43	89
	Negative	0	9

Results from qPCR and normalized OD values from days -1, 2, 4, 6, 19–24 and 41–55. Out of 141 samples, 52 were concordant (43 samples tested positive, and 9 samples tested negative by both methods). All ELISA positive samples were also positive by qPCR.

All 43 ELISA-positive samples are also qPCR positive, while most (89/132) of the qPCR-positive samples are ELISA negative (Table 2). While in the absence of a gold standard, we cannot know the true *Cryptosporidium* infection status of a given sample, this result implies that ELISA was at best as sensitive, but more likely less sensitive than qPCR to detect *Cryptosporidium* in our sample set. ELISA was, however, at least as specific for *Cryptosporidium* as qPCR since all 9 negative qPCR samples are also negative with ELISA (Table 2). Further, computing coefficients of variation and coefficients of quartile variation, ELISA was the more variable measurement, both when calculated across all samples, within individuals or across individuals (see S1 Table in S1 File).

Baseline samples (study day minus 1) for each participant were examined by both plate- and array card-based qPCR, the latter of which was used to detect enteric pathogens in addition to *Cryptosporidium*. As shown in Table 3, 100% of enrolled patients (n = 22) had *Cryptosporidium* detected by both plate-based and array card-based qPCR with an average 1.35 difference in C_t_ values between the two PCR formats, with the plate-based PCR usually having the higher value. TAC PCR confirmed that many of our study participants were infected with enteric pathogens in addition to *Cryptosporidium*, but not necessarily in diarrheagenic amounts (*i*.*e*. pathogen load in a patient at which the pathogen is most likely contributing to diarrhea; see below). Amongst these were Shigella (or enteroinvasive *Escherichia coli*), Norovirus GII, and *Campylobacter jejuni/coli*. Utilizing PCR C_t_ cutoffs previously developed for the Global Enteric Multisite Study (GEMS) [28] we assessed detected pathogens for amounts considered diarrheagenic (See S2 Table in S1 File for cutoff values). As shown in Table 3 (TAC-detected co-pathogens), we noted 7 *Cryptosporidium* detections (below C_t-_ 24.0, 5 in the treatment group and 2 in the placebo group) and 4 heat stable toxin-producing enterotoxigenic *E*. *coli* detections (below C_t_ 22.8 (ST-ETEC; 3 in the treatment group, 1 in the placebo group) at diarrheagenic amounts. Of note, these two pathogens were not detected together wherein both pathogens were detected at diarrheagenic amounts.

**Table 3:** For each participant in Part A of the CRYPTOFAZ study, the results from all DNA-based assays are displayed, including the plate-based *Cryptosporidium* 18s qPCR C_t_ value for the Day 0 first sample of the day, the array card-based *Cryptosporidium* 18s qPCR C_t_ value for the Day 0 first sample of the day, species and subtyping results from dideoxysequencing, and the co-pathogens detected through the array card (C_t_ value listed in parentheses).

Subject ID	Plate *Crypto*. C_t_	TAC *Crypto* C_t_.	*Crypto*. Species detected	gp60-based subtype	TAC-detected co-pathogens
CFZ01	24.6	29.8	*viatorum*	XVaA3	*Campylobacter* pan (32.7), *E*. *bieneusi* (26.9), EAEC (25.7), ST-ETEC (22.6)
CFZ02	29.3	21.8	*parvum*	IIcA5G3	EAEC (33.7), *Shigella*/EIEC (32.4)
CFZ03	28.2	24.2	*hominis*	IeA11G3T3	Blastocystis (33.8), ST-ETEC (34.8), Sapovirus (34.8)
CFZ04	20.5	23.1	*parvum*	IIcA5G3	*E*. *bieneusi* (23.0), Norovirus GII (28.0)
CFZ05	31.8	28.0	*parvum*	n/a	Blastocystis (34.6), *Campylobacter* jejuni/coli (33.0), *Campylobacter* pan (30.9), *E*. *bieneusi* (26.7), EAEC (24.7), ST-ETEC (21.3), *H*. *pylori* (34.6)
CFZ06	28.2	33.0	*parvum*	IIcA5G3	Adenovirus pan (32.3), *E*. *bieneusi* (29.1), EAEC (30.2), aEPEC (30.4), ST-ETEC (18.6)
CFZ07	28.9	26.0	*hominis*	IdA20	*Campylobacter jejuni/coli* (26.0), *Campylobacter* pan (24.3), LT-ETEC (25.9)
CFZ08	30.2	27.9	*unknown*	n/a	Adenovirus pan (33.5), *E*. *bieneusi* (33.7), EAEC (21.0), aEPEC (28.9), *Shigella*/EIEC (33.2)
CFZ09	30.2	22.8	*parvum*	IIcA5G3	Blastocystis (32.6), EAEC (31.3), aEPEC (24.5), LT-ETEC (33.8), *Giardia* (31.1)
CFZ10	26.9	20.9	*meleagridis*	IIIdA6	*E*. *bieneusi* (25.5)
CFZ11	22.5	23.1	*parvum*	IIcA5G3	*B*. *fragilis* (29.9), aEPEC (22.9), ST-ETEC (33.3), Salmonella (34.0)
CFZ12	30.1	32.6	*parvum*	IIcA5G3	Adenovirus 40/41 (33.3)
PCB01	29.1	31.7	*parvum*	IIcA5G3	Astrovirus (25.6), EAEC (15.0), LT-ETEC (32.7), *Giardia* (34.3), Norovirus GII (25.5)
PCB02	26.3	16.8	*unknown*	n/a	EAEC (24.0), aEPEC (28.9), ST-ETEC (34.2), *Giardia* (28.2),
PCB03	28.5	26.6	*hominis*	n/a	EAEC (22.5), LT-ETEC (31.8), *Giardia* (33.5), *Shigella*/EIEC (32.4)
PCB04	26.8	32.7	*meleagridis*	IIIdA6	EAEC (22.9), ST-ETEC (33.0), *Giardia* (33.4), *Shigella*/EIEC (33.6)
PCB05	23.3	23.1	*parvum*	IIcA5G3	EAEC (18.7)
PCB06	28.7	24.7	*meleagridis*	IIIdA6	EAEC (30.2), aEPEC (22.0), ST-ETEC (17.5)
PCB07	29.5	26.8	*unknown*	n/a	*E*. *bieneusi* (26.0)
PCB08	29.5	27.9	*parvum*	IIcA5G3	Adenovirus pan (30.9), *Campylobacter* pan (31.9)
PCB09	26.7	30.3	*meleagridis*	IIIdA6	Blastocystis (33.4), EAEC (33.2), aEPEC (25.4), Entamoeba pan (29.8)
PCB10	29.7	25.9	*parvum*	IIcA5G3	Blastocystis (32.9), E. bieneusi (25.2), EAEC (24.1), aEPEC (33.9), ST-ETEC (34.2), *Giardia* (33.8), Sapovirus (25.2), *Shigella*/EIEC (28.5)

CFZ, participant randomized to the clofazimine arm of the study; PCB, participant randomized to the placebo arm of the study; TAC, TaqMan Array Card; EAEC, enteroaggregative *E*. *coli*; EHEC, enterohemorrhagic *E*. *coli*, EIEC, Enteroinvasive *E*. *coli*; aEPEC, atypical enteropathogenic *E*. *coli*, tEPEC, typical enteropathogenic *E*. *coli*; LT-ETEC, heat labile toxin-producing enterotoxigenic *E*. *coli*; ST-ETEC, heat stable toxin-producing enterotoxigenic *E*. *coli*; n/a, subtype undetected.

Baseline samples for our participants were analyzed to determine their *Cryptosporidium* species and subtypes using commercial sequencing. As shown in Table 3, half of all identifiable infections were caused by *C*. *parvum*. Sequencing of the gp60 gene revealed that 9 of these belonging to family IIcA5G3, a family of *C*. *parvum* previously observed in other human infections [29, 30] (See S3 Table in S1 File for NCBI accession numbers for 18S rRNA and gp60 sequences). Also identified were *C*. *meleagridis* and *C*. *viatorum*.

## 4.0 Discussion

The CRYPTOFAZ trial was conducted in response to the unmet need for new *Cryptosporidium* treatments in the HIV population. For the screening/recruitment and for monitoring of oocysts shedding during the trial period, several different diagnostic methodologies were employed for the detection and characterization of *Cryptosporidium*, namely RDTs, ELISA, both plate- and array card-based qPCR, and *Cryptosporidium* subtyping.

Both RDTs and plate-based qPCR were used to screening potential participants, this was done to take advantage of the combined specificity of these methods to exclude those patients who were experiencing diarrhea for reasons excluding cryptosporidiosis. The use of qPCR for this purpose proved quite advantageous, owing to its increased sensitivity over rapid diagnostic tests for *Cryptosporidium* detection, and is consistent with previous research conducted in Malawi in a study employing RDTs and PCR [31]. Of the 75 potential *Cryptosporidium*-positive individuals (*i*.*e*. those that were qPCR positive) considered for the trial, >70% (n = 54, Table 1) were considered for participation based on the qPCR result alone. None of these individuals would have been considered had only an RDT been performed for screening/recruitment.

Both ELISA and plate-based qPCR were used to monitor daily oocyst shedding during the in-patient period and two follow-up visits to determine if those study participants receiving CFZ cleared *Cryptosporidium* more effectively versus participants randomized to placebo. In our comparative analysis of the two methods, we saw good qualitative agreement between the two methods, but noted that overall, ELISA was less sensitive and more variable. It is generally acknowledged that qPCR possesses higher sensitivity to detect the presence of bacteria as compared to ELISA [17, 18]. And in our analysis, it was also the method with lower variability. However, qPCR results can be affected by the likely persistence of *Cryptosporidium* nucleic acid in the gut 40 days after the initial detection [19].

The use of TAC allowed us to further confirm *Cryptosporidium* infections and detect other co-pathogens. Examining the C_t_ values for all enteric pathogens detected allowed us to conclude that for most participants, *Cryptosporidium* appeared to be the pathogen of highest abundance including 7 instances (5 in the CFZ treatment group and 2 in those receiving placebo) where it was detected with a C_t_ value below what is highly associated with diarrhea, giving us confidence that screening protocols had worked as intended. However, while *Cryptosporidium* was detected in the baseline samples of all our participants, in 4 participants the likely major contributor to their diarrhea was ST-ETEC rather than *Cryptosporidium*. The finding that *Cryptosporidium* was the most abundant pathogen in stool samples of our study population is consistent with findings of Carcamo et al [34] in Peru who found that *Giardia lamblia* and /or *Cryptosporidium* was strongly associated with Diarrhea among HIV infected individuals [32].

Given that our data demonstrates that our population all had multiple enteric infections in addition to *Cryptosporidium*, we would submit that if budget allows, future investigators carrying out similar research should consider conducting an initial screening for multiple pathogens among diarrhea patients. This would allow researchers to know which pathogens are present and determine whether or not cryptosporidium is likely a major contributor to the patients’ diarrhea before conducting cryptosporidium ELISA or PCR on follow up samples.

Using both RDT and plate-based qPCR at screening (though study inclusion was based on the qPCR result) was advantageous for identifying potential trial participants. However, use of TAC on baseline samples allowed us to identify that only 7 of 22 participants had *Cryptosporidium* in diarrheagenic amounts by using the GEMS study cut-off. As this was a population that was targeted for drug efficacy evaluation according to the trial protocol, future researchers may need to take this phenomenon into account when conducting similar studies.

We employed Sanger sequencing to determine the *Cryptosporidium* species and subtypes. Unsurprisingly, the majority of contributing infections involved *C*. *parvum*. Further, amongst those *C*. *parvum* infections, gp60 sequencing revealed that 10 of 11 infections were of family *IIc*. This subtype is thought to be anthroponotic and it has been previously observed in other *Cryptosporidium* infection clusters. However, the remaining 5 identifiable infections were shown to be comprised of *C*. *meleagridis* and *C*. *viatorum*. At time of writing, to our knowledge this is the first documented case of *C*. *viatorum* in Southern Africa, having only been previously documented in Ethiopia and Nigeria [33–35].

**Fig 2:**
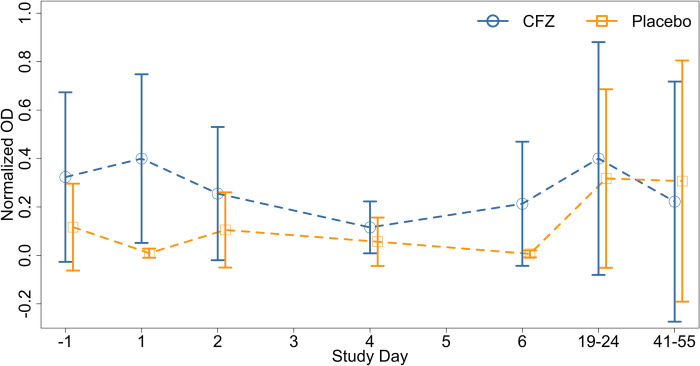
Mean normalized ELISA optical density over time. Normalized OD values from days -1, 1, 2, 4, 6, 19–24, and 41–55 were averaged according to participant randomization to the CFZ or placebo arms of the trial. Circle markers—participants randomized to CFZ; square markers—participants randomized to placebo.

We are mindful that CRYPTOFAZ was designed to test the efficacy of CFZ and not explicitly designed to test the robustness of the diagnostics. Because of this, the analysis presented in the current work has a few limitations. Samples collected during the inpatient and follow-up visits were frozen prior to batch processing and testing in ELISA and qPCR. Because these samples underwent a freeze-thaw cycle, it may be possible that this may have led to some loss in sensitivity in both diagnostics. In addition, single concentration positive controls were used in ELISA rather than fitted standard curves as were employed in qPCR. Having an oocyst standard curve for ELISA derived from the same oocyst source used for Cryptosporidium genomic DNA would have allowed us to perform more direct comparisons of sensitivity and detection between the diagnostics. Our analyses of the ELISA as presented in Figs 1A and 2 rely on normalized optical density values. This amounts to a quantitative analysis, which this particular ELISA test was not designed/validated for. We should note that our use of C_t_ cutoffs for determination of diarrheagenic amounts of enteric pathogen should be interpreted with caution. The C_t_ cutoffs were developed as part of the GEMS study whose participants were children, who had moderate to severe diarrhea and a significant proportion of children were immunocompetent as opposed to our study participants who were immunocompromised adults with persistent diarrhea. Unfortunately, no similar C_t_ cutoffs have been developed for use with adult HIV populations. in addition, we genotyped samples from study day minus 1 only, there the possibility that subtypes shifted between the initial week (day -1 to day 6) and the day 19–24 and day 41–55 follow-up visits.

In summary, this study demonstrates that ELISA and qPCR can demonstrate differences between groups under study for cryptosporidium. The limitation of the ELISA is the numbers of subjects are much less than can be detected with qPCR. However, the relative ease of ELISA may outweigh the need for enrolling high numbers of subjects in certain groups where cryptosporidiosis is of high prevalence.

## Supporting information

S1 File The supplementary information file contains 1 supplementary figure (S1 Fig: log2 oocyst per gran vs. qPCR Ct values plot) and 3 supplementary tables (S1 Table: overall, intra- and inter-individual coefficients of variation (CV) and coefficient of quartile variation (CQV) for ELISA and qPCR; S2 Table: qPCR cut-offs; S3 Table: NCBI accession numbers for 18s and gp60 sequences). (DOCX)
