# Supplementary material for: Examination of ELISA against PCR for assessing treatment efficacy against Cryptosporidium in a clinical trial context
Source: PLoS One. 2023 Sep 8;18(9):e0289929. doi: 10.1371/journal.pone.0289929 (PMC10490871; doi:10.1371/journal.pone.0289929)
Supplement: S1 File — (DOCX) [file pone.0289929.s001.docx]

**Examination of *Cryptosporidium* Diagnostic Performance for Assessing**

**Treatment Efficacy in a Clinical Trial Context**

James T. Nyirenda^1*^, Marc Y. R. Henrion^1,2*^, Vita Nyasulu^1^, Mike Msakwiza^1^, Wilfred Nedi^1^, Herbert Thole^1^ Jacob Phulusa^1^, Neema Toto^1^, Khuzwayo C. Jere^1,3^, Alex Winter^4^ , Leigh A. Sawyer^4^, Thomas Conrad^4^, Donnie Herbert^4^, Crystal Chen^4^, Wesley C. Van Voorhis^6^, Eric R. Houpt^5^, Pui-Ying Iroh Tam^1,2^, Darwin J. Operario^5^

*Authors contributed equally to this work

^1^Malawi-Liverpool-Wellcome Trust Clinical Research Programme, Blantyre, Malawi

^2^Liverpool School of Tropical Medicine, Liverpool, UK

^3^Centre for Global Vaccine Research, Institute of Infection, Veterinary and Ecological Sciences, University of Liverpool, Liverpool, UK

^4^Emmes Corporation, Rockville, Maryland, USA

^5^University of Virginia, Charlottesville, Virginia, USA

^6^University of Washington, Seattle, Washington, USA

**S1 Figure**

**log2 oocyst per gram versus qPCR Ct values plot**. This is an inverse relationship between log 2 oocyst count and qPCR Ct value, each experimental batch had standard curve for samples from all visits per participant. Cryptosporidium Ct value was converted to a genome count by comparison to the standard curve. Genome counts were then converted to oocyst counts.

**
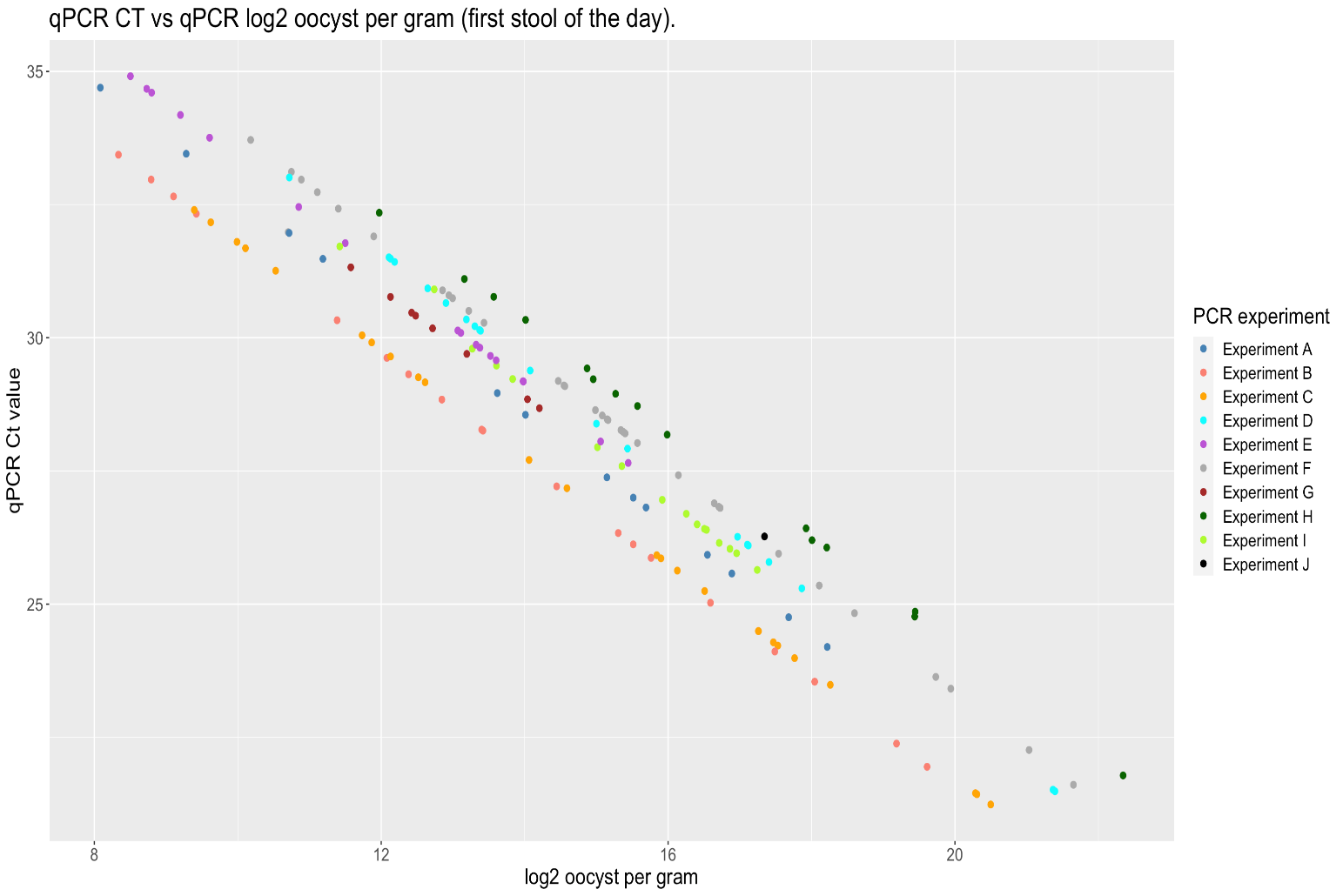
**

**S1Table**

**Overall, intra- and inter-individual coefficients of variation (CV) and Coefficient of quartile variation (CQV)**. Given that the CV requires a meaningful zero point. We used 1/Ct for the qPCR measurements in these calculations and unnormalised OD score

|  | **Overall** | **Intra-individual** | **inter-individual** |
| --- | --- | --- | --- |
| **CV** |  |  |  |
| qPCR | 0.1334 | 0.0941 | 0.0927 |
| ELISA | 1.9515 | 1.2671 | 1.3849 |
| **CQV** |  |  |  |
| qPCR | 0.0885 | 0.0481 | 0.0469 |
| ELISA | 0.9861 | 0.6367 | 0.9895 |

**S2 Table**

The table below is adapted from Liu et al.(1), Supplementary Table S4 represents qPCR cutoffs for a pathogen being detected in a “highly diarrhea-associated quantity” from the TaqMan Array Card reanalysis of samples from the Global Enteric Multisite Study (“GEMS”). Each cutoff is defined as the quantity (as defined by qPCR C_t_) at which the point estimate of the odds ratio exceeded 2. If the C_t_ “value is at or below this cutoff, the pathogen was detected at an amount considered to be highly diarrheagenic. Cutoffs were not calculated for all possible pathogens in the GEMS study. Cutoffs are displayed below only for those pathogens identified through array card analysis in the CRYPTOFAZ study.

| Pathogen | Quantification Cutoff (C_t_) |
| --- | --- |
| *Shigella*/EIEC | 27.9 |
| *Salmonella* | 30.7 |
| ST-ETEC (STh) | 22.8 |
| *Helicobacter pylori* | 30.8 |
| Astrovirus | 22.2 |
| *Cryptosporidium* | 24.0 |
| Norovirus GII | 23.4 |
| Adenovirus 40/41 | 22.7 |
| *C. jejuni/C. coli* | 15.4 |
| Typical EPEC (*bfpA*) | 16.0 |

**S3 Table**

NCBI accession numbers for 18s and gp60 sequences

| Participant ID | 18s | gp60 |
| --- | --- | --- |
| CFZ01 | MW147226 | MW159770 |
| CFZ02 | N/A | MW159771 |
| CFZ03 | N/A | MW159772 |
| CFZ04 | N/A | MW159773 |
| CFZ05 | N/A | N/A |
| CFZ06 | MW147227 | MW159774 |
| CFZ07 | MW147228 | MW159775 |
| CFZ08 | N/A | N/A |
| CFZ09 | N/A | MW159776 |
| CFZ10 | N/A | MW159777 |
| CFZ11 | MW147229 | MW159778 |
| CFZ12 | N/A | MW159779 |
| PCB01 | N/A | MW159780 |
| PCB02 | N/A | N/A |
| PCB03 | N/A | N/A |
| PCB04 | N/A | MW159781 |
| PCB05 | MW147230 | MW159782 |
| PCB06 | N/A | MW159783 |
| PCB07 | N/A | N/A |
| PCB08 | N/A | MW159784 |
| PCB09 | N/A | MW159785 |
| PCB10 | N/A | MW159786 |

**References**

1. Liu J, Platts-Mills JA, Juma J, Kabir F, Nkeze J, Okoi C, et al. Use of quantitative molecular diagnostic methods to identify causes of diarrhoea in children: a reanalysis of the GEMS case-control study. Lancet. 2016 Sep 24;388(10051):1291–301.
